# Lymphocytic Interstitial Pneumonia in a Man with Human Immunodeficiency Virus Infection

**DOI:** 10.1590/0037-8682-0055-2023

**Published:** 2023-06-02

**Authors:** Merve Nur Tasdemir, Serdar Aslan

**Affiliations:** 1 Giresun University, Faculty of Medicine, Department of Radiology, Giresun, Turkey.

Lymphocytic interstitial pneumonia (LIP) is a benign lung disorder characterized by diffuse lymphocytic infiltration and polyclonal parenchymal proliferation^1^. Lymphoid interstitial pneumonia (LIP) is a rare disease that can occur in individuals with human immunodeficiency virus (HIV) infection. Less than 5% of HIV-positive adults are affected^2^ .

**FIGURE 1:**
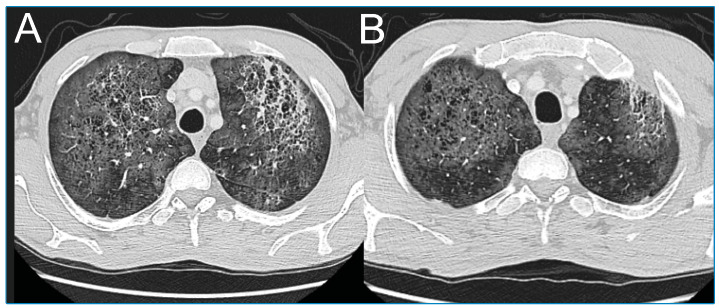
**(A-B):** Thoracic computed tomography showing ground-glass areas accompanied by multiple millimeter-sized thin-walled cysts and cystic changes in both lungs.

**FIGURE 2:**
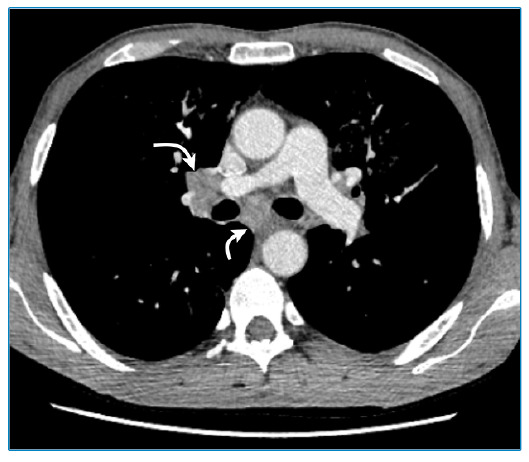
Thoracic computed tomography showing mediastinal lymphadenopathy (curved arrows).

A 52-year-old man was admitted to the internal medicine department because of weight loss. Serological testing revealed that he was HIV positive. Thoracic computed tomography (CT) was performed because he reported having flu-like symptoms for one month. Ground-glass opacities, accompanied by millimeter-sized thin-walled cysts, were observed in both lungs, which were more prominent in the upper lobes (Figure 1). Mediastinal lymphadenopathy was also present (Figure 2).

Approximately 5% of LIP cases undergo malignant transformation to lymphoma^2^. The treatment of LIP differs from that of other opportunistic infections because it is generally responsive to steroids^1^ ^-^ ^2^. Therefore, it should be considered in the differential diagnosis of HIV-positive patients with respiratory complaints. Specific imaging characteristics such as thin-walled cysts in areas of ground-glass opacities can help to make the diagnosis.
